# Dr. Delevan E. Blackman

**Published:** 1909-12

**Authors:** 


Dr. Delevan E. Blackman, of Buffalo, died at his home, No. 541 North Division street, November 12, 1909. He graduated at the Medical Department, University of Buffalo, in 1878. He was a practitioner here for many years. His death resulted from pneumonia.

